# Selection in action: dissecting the molecular underpinnings of the increasing muscle mass of Belgian Blue Cattle

**DOI:** 10.1186/1471-2164-15-796

**Published:** 2014-09-17

**Authors:** Tom Druet, Naima Ahariz, Nadine Cambisano, Nico Tamma, Charles Michaux, Wouter Coppieters, Carole Charlier, Michel Georges

**Affiliations:** Unit of Animal Genomics, GIGA-R & Faculty of Veterinary Medicine, University of Liège – B34 (+1), Avenue de l’Hôpital 1, Liège, B-4000 Belgium; FARAH & Faculty of Veterinary Medicine, University of Liège (B43), Liège, Belgium; Faculty of Medicine, Université Libre de Bruxelles (CP 619), Brussels, Belgium

**Keywords:** Selective sweeps, Muscular development, Association studies, Cattle, Polygenic architecture, Genetic architecture, Complex traits

## Abstract

**Background:**

Belgian Blue cattle are famous for their exceptional muscular development or “double-muscling”. This defining feature emerged following the fixation of a loss-of-function variant in the myostatin gene in the eighties. Since then, sustained selection has further increased muscle mass of Belgian Blue animals to a comparable extent. In the present paper, we study the genetic determinants of this second wave of muscle growth.

**Results:**

A scan for selective sweeps did not reveal the recent fixation of another allele with major effect on muscularity. However, a genome-wide association study identified two genome-wide significant and three suggestive quantitative trait loci (QTL) affecting specific muscle groups and jointly explaining 8-21% of the heritability. The top two QTL are caused by presumably recent mutations on unique haplotypes that have rapidly risen in frequency in the population. While one appears on its way to fixation, the ascent of the other is compromised as the likely underlying *MRC2* mutation causes crooked tail syndrome in homozygotes. Genomic prediction models indicate that the residual additive variance is largely polygenic.

**Conclusions:**

Contrary to complex traits in humans which have a near-exclusive polygenic architecture, muscle mass in beef cattle (as other production traits under directional selection), appears to be controlled by (i) a handful of recent mutations with large effect that rapidly sweep through the population, and (ii) a large number of presumably older variants with very small effects that rise slowly in the population (polygenic adaptation).

**Electronic supplementary material:**

The online version of this article (doi:10.1186/1471-2164-15-796) contains supplementary material, which is available to authorized users.

## Background

**Figure 1:**
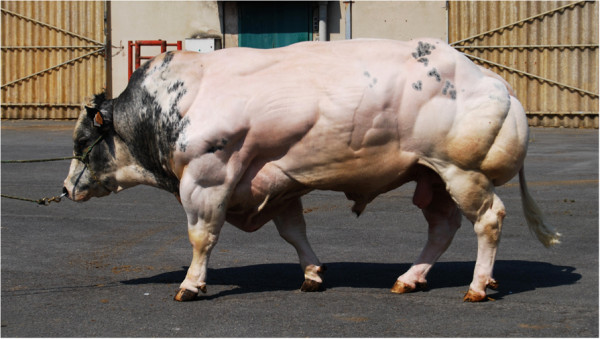
**Muscular development of a typical sire from the modern Belgian Blue Cattle (BBC) breed (Passe-Partout).**

Belgian Blue Cattle (BBC) are famous for their exceptional muscular development referred to as “double-muscling” (Figure 1). BBC derive from a dual-purpose breed that roamed Southern Belgium in the beginning of the twentieth century. Although a small population (~3,400) of dual-purpose animals corresponding to this ancestral “*Race de Moyenne et Haute Belgique*” still exists (now referred to as “*Blancs Bleus Mixtes*” or BBM), intense selection for increased muscle mass, driven by premiums paid for double-muscled carcasses and enabled by the systematization of artificial insemination (AI) in the sixties, led to the fixation - in less than 20 years (i.e. ~5 generations) - of what has become the defining feature of the new BBC breed. It was demonstrated (f.i. [1]) that the rapid metamorphosis that affected BBC between ~1960 and ~1985 was largely due to the fixation of a loss-of-function mutation (*p.D273RfsX13*) in the myostatin (*MSTN*) gene. *MSTN* encodes a “chalone” (i.e. a hormone inhibiting the growth of the tissue by which it is produced) controlling the growth of skeletal muscle in wild-type individuals (f.i. [2, 3]).

It is generally not appreciated, however, that (i) the heritability of muscular development remained as high as 30-45% in BBC after fixation of the *p.D273RfsX13 MSTN* allele, and (ii) that this residual genetic variation has been exploited to further increase muscle mass of BBC. That homozygosity for the *p.D273RfsX13 MSTN* variant only accounts for part of the spectacular muscular hypertrophy of modern BBC is also obvious from the comparison of their carcass scores with that of present-day BBM animals that are homozygous for the *p.D273RfsX13 MSTN* mutation (Additional file 1: Figure S1).

In this paper, we aimed to define the nature of the genetic determinants underpinning the second wave (after 1985) of muscle growth witnessed in BBC. Did it involve the rapid fixation of one or more mutations with effects of a magnitude similar to the *p.D273RfsX13 MSTN* mutation? Are Quantitative Trait Loci (QTL) with large effects on muscle mass segregating in present-day BBC? Or is the residual heritability for muscle mass in present-day BBC largely polygenic or “quasi-infinitesimal”?

## Results

**Figure 2:**
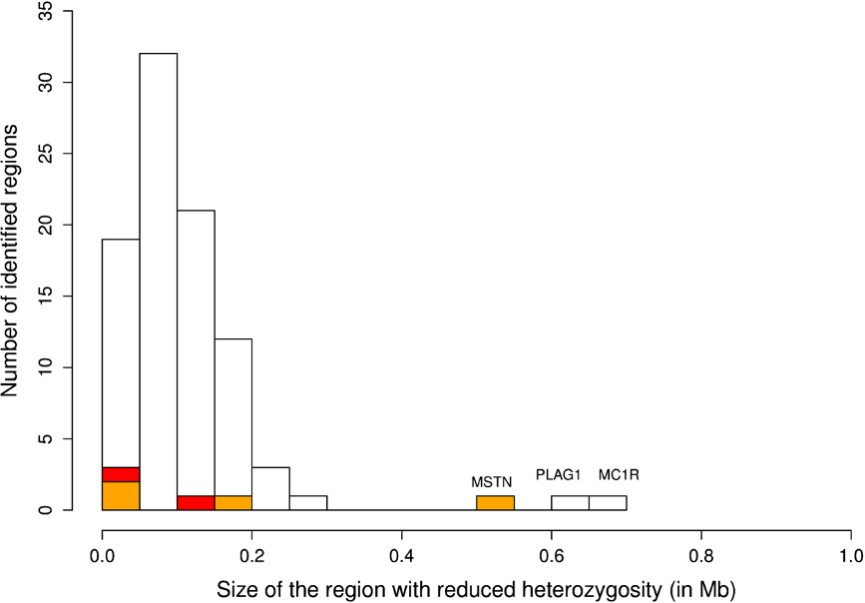
**Distribution of sweep sizes.** Size (in megabases) of 91 genomic regions with reduced genetic variability in modern BBC animals (born after 1999). Regions differentiated with respect to both BBM and HF are in orange whereas red segments represent regions differentiated exclusively with respect to HF.

To address the first question (i.e. ***did the second wave of muscle growth that occurred in BBC after 1985, involve the rapid fixation of variants with major effect on muscle mass***, hence mimicking the fixation of the *p.D273RfsX13 MSTN* mutation prior to 1985?) we aimed at identifying genomic regions characterized by (i) reduced genetic variability (as a result of a selective sweep) in “modern” BBC (born in 2000 or later), and (ii) high genetic differentiation (F_ST_-based measure; see M&M) with “old” BBC (born in 1985 or before), BBM, and Holstein-Friesian (HF) animals. HF were selected as controls because they are of dairy type, yet are phylogenetically closely related to BBC (Additional file 2: Figure S2). We genotyped (i) 301 “modern” BBC, (ii) 28 “old” BBC (all homozygous for the *p.D273RfsX13 MSTN* mutation), (iii) 52 animals from the dual-purpose BBM subpopulation, and (iv) 191 Dutch HF sires, with the Illumina BovineHD array interrogating >700 K single nucleotide polymorphisms (SNP) dispersed across the genome. We first identified regions of low heterozygosity using a previously reported Hidden Markov Model (HMM) implemented with the “SWEEPY” program [4]. We identified a total of 91 candidate regions exhibiting reduced variability in modern BBC (Additional file 3: Table S1). Three of these departed clearly from the bulk by their larger size (Figure 2): (i) a 692 Kb segment on chromosome BTA18 spanning the *Extension* locus (*MC1R*) affecting coat color [5], (ii) a 609 Kb segment on BTA14 encompassing *PLAG1* known to affect stature in cattle [6], and (iii) a 504 Kb segment on BTA2 containing *MSTN* (Additional file 4: Figure S3, Additional file 5: Figure S4 and Additional file 6: Figure S5). The *MC1R* and *PLAG1* regions were also characterized by signatures of reduced variability in old BBC, BBM and HF (the same haplotype being fixed in the four studied populations). The corresponding signatures are assumed to be genuine and to result from selection for black-based coat color and increased stature. The *MSTN* region exhibited a signature of reduced variability in old BBC, but not in BBM and HF. The region was highly differentiated between BBC on the one hand, and BBM and HF on the other hand. This was exactly as expected for a selective sweep accompanying the dissemination of double-muscling in BBC as a result of the fixation of the *p.D273RfsX13 MSTN* variant. Of the 88 remaining (smaller) regions, only three were differentiated with respect to BBM and HF, and two with respect to HF only (Additional file 3: Table S1). However, none of the candidate regions exhibited noticeable levels of differentiation with old BBC, BBM and HF, as would be expected for a genuine selective sweep that would have occurred in modern BBC. Taken together, these results do not support the fixation of an allele with major effect on muscularity in BBC during the second wave of muscle growth.

To provide a response to the second question (i.e. ***are there QTL with large effects on muscularity segregating in BBC*?**), we performed a genome-wide association study (GWAS) for muscularity in modern BBC. In addition to the 28 old and 301 modern BBC sires used to detect signatures of selection (*vide supra*), we genotyped 264 sires born between 1986 and 1999 with the Illumina BovineHD array, for a total of 593 genotyped BBC sires. All were AI sires and 555 of them had recorded offspring (304 on average and ranging from 1 - 4675). The phenotypes used for GWAS were twice the “Daughter Trait Deviations” (DTD) for five categorical traits pertaining to muscular development: general muscularity (GM), muscularity of the back (BM), muscularity of the shoulder (SM), muscularity of the rump (rear view) (RMR), and muscularity of the rump (side view) (RMS). Association analyses were conducted using a previously described haplotype-based method [7].

**Figure 3:**
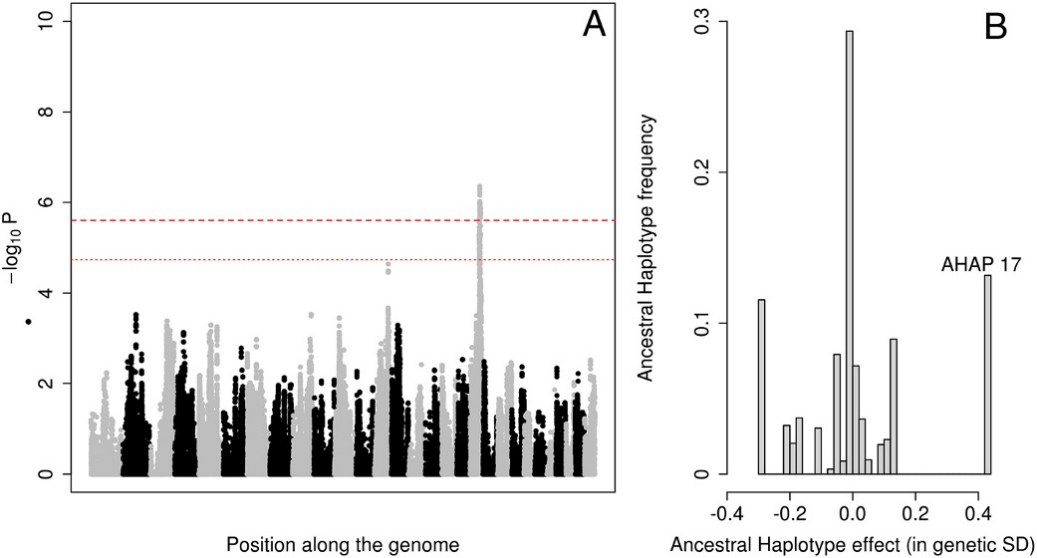
**Genome scan for muscularity of the back. A**. Manhattan plot for BM (muscularity of the back). Alternating gray and black symbols mark the limits between successive chromosomes. The two red horizontal lines correspond to the thresholds for genome-wide significant and suggestive association, respectively. **B**. Effect (X-axis) and population frequency (Y-axis) of the 20 ancestral haplotypes fitted in the association model.

The likelihood ratio test (LRT) exceeded the genome-wide significance threshold (-log_10_p > 5.60) at a single genomic location (BTA19, 46.6-48.0 Mb interval) for GM and BM. The same chromosomal region clearly affected SM, RMR and RMS as well, although the corresponding effects were not genome-wide significant. The observed signal was primarily driven by one haplotype (AHAP17) increasing muscle mass. The QTL explained between 4.5% (RMS) and 7.8% (BM) of the total additive genetic variance (estimated as the variance of the polygenic effects in a model without haplotype effects) (Figure 3, Table 1, Additional file 7: Figure S6, Additional file 8: Figure S7, Additional file 9: Figure S8, Additional file 10: Figure S9 and Additional file 11: Figure S10). We noticed that the most likely positions of the QTL flanked the *MRC2* gene (BTA19, ~47.7 Mb). We previously reported that loss-of-function mutations (*c.2904-2905delAG*, *c.1906 T > C*) in the *MRC2* gene cause Crooked-Tail Syndrome (CTS) in homozygotes/compound heterozygotes and are associated with increased muscle mass and decreased height in heterozygotes in BBC [8, 9]. Of note, AHAP17 was likewise associated with a decrease in height in our dataset (data not shown). We genotyped the 593 BBC sires for the corresponding *MRC2* mutations. Twenty four and 0.2 percent of the bulls were carriers for the *c.2904-2905delAG* and *c.1906 T > C* mutations, respectively, in agreement with previous estimates. Linkage disequilibrium between AHAP17 and the *c.2904-2905delAG* variant was 0.905 (*r*^*2*^) or 0.992 (*D’*). This indicates that the more common *c.2904-2905delAG* mutation is virtually exclusively encountered on the AHAP17 haplotype, but that mutation-free versions of the latter also exist. We added *MRC2* genotype to the association model and showed that it had a major effect on all muscularity phenotypes as expected (Additional file 12: Table S2). Statistical significance (-log_10_p) of the residual haplotype effects in the BTA19 46.6-48.0 Mb interval dropped severely for GM, BM, and SM, but remained as high as 3.53 and 2.79 for RMR and RMS. This suggests that the *MRC2* variants accounts for most if not the entire QTL effect on GM, BM and SM, but that other variants with more modest effects on RMR and RMS may exist in this chromosomal region (Additional file 7: Figure S6, Additional file 8: Figure S7, Additional file 9: Figure S8, Additional file 10: Figure S9 and Additional file 11: Figure S10, Additional file 13: Figure S11, Additional file 14: Figure S12, Additional file 15: Figure S13, Additional file 16: Figure S14 and Additional file 17: Figure S15).

**Table 1:** **Identified suggestive and genome-wide significant QTLs and their associated variance (in % of additive genetic variance)**

Chromosome	Position	Trait	Model1 ^1^-log10(p-value)	Model2 ^2^-log10(p-value)	Proportion of genetic variance (in %)
2	52.612052	RMS	5.24	5.36	6.2
	52.612052	RMR	5.27	5.39	6.6
9	92.482709	SM	5.24	5.24	7.6
14	62.670140	RMR	NS	4.78	6.3
	62.653097	RMS	4.91	4.97	6.5
19	7.848400	GM	NS	**5.66** ^**3**^	7.2
	8.363013	BM	NS	3.78^4^	4.5
	7.848400	SM	NS	4.71^4^	5.9
	8.526105	RMR	5.06	**5.64** ^**3**^	5.9
	7.786912	RMS	5.25	**5.85** ^**3**^	6.4
19	46.589690	GM	**5.72** ^**3**^	NS	7.1
	47.966715	BM	**6.36** ^**3**^	NS	7.8
	46.594963	SM	4.59^4^	NS	5.3
	46.468971	RMR	4.78	NS	5.4
	46.468971	RMS	3.59^4^	NS	4.5

^1^Initial scan (without the *MRC2* genotype effect).

^2^Second scan with the *MRC2* genotype fitted as a fixed effect.

^3^Genome-wide signicant QTL (in bold).

^4^Summaries of QTLs reaching genome-wide significance for at least one trait were reported for all traits.

**Figure 4:**
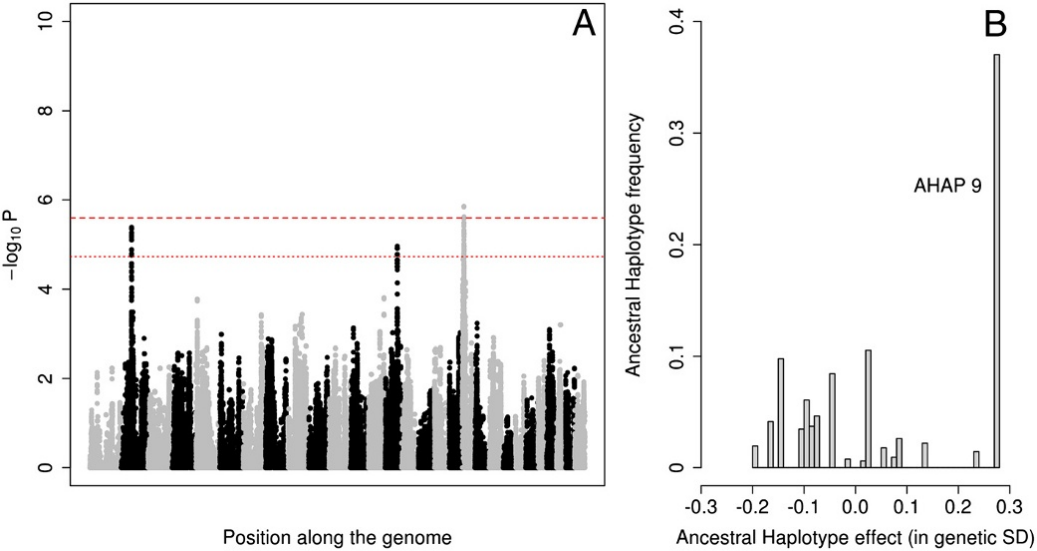
**Genome scan for muscularity of the rump. A**. Manhattan plot for RMS (muscularity of the rump – side view) obtained when including *MRC2* genotype in the model. Alternating gray and black symbols mark the limits between successive chromosomes. The two red horizontal lines correspond to the thresholds for genome-wide significant and suggestive association, respectively. **B**. Effect (X-axis) and population frequency (Y-axis) of the 20 ancestral haplotypes fitted in the association model.

In addition to the BTA19 QTL, the initial genome scan revealed four signals exceeding the genome-wide suggestive threshold (-log_10_p > 4.73), respectively on chromosomes BTA2 (52.6 Mb), BTA9 (92.5 MB), BTA14 (62.6 Mb) and BTA19 (7.8 Mb) (Table 1). We rescanned the entire genome, including *MRC2* genotype in the model. The second BTA19 QTL (7.8-8.5 Mb interval) became genome-wide significant for GM, RMR and RMS. The effect was largely driven by a single haplotype (AHAP9) increasing muscle mass. It explained between 4.5% (BM) and 7.2% (GM) of the additive genetic variance (Figure 4, Table 1, Additional file 7: Figure S6, Additional file 8: Figure S7, Additional file 9: Figure S8, Additional file 10: Figure S9 and Additional file 11: Figure S10, Additional file 13: Figure S11, Additional file 14: Figure S12, Additional file 15: Figure S13, Additional file 16: Figure S14 and Additional file 17: Figure S15). The remaining three QTL remained suggestive (Table 1).

**Figure 5:**
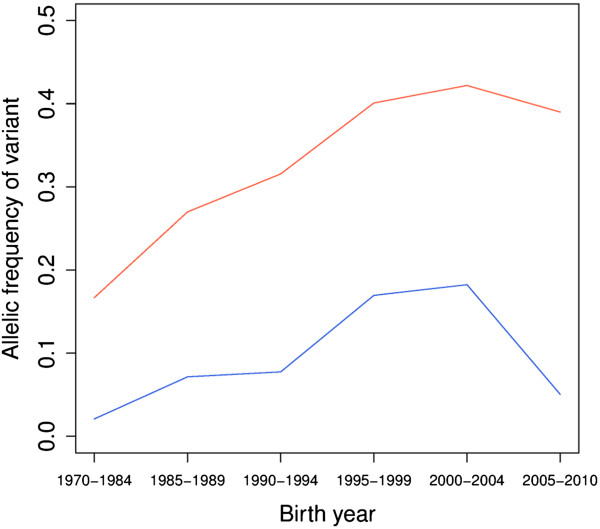
**Evolution of the allelic frequency of significant variants.** Evolution of the frequency of the *c.2904-2905delAG* variant (in red) in *MRC2* (causing the Crooked-Tail Syndrome) and the AHAP9 haplotype (in blue) related to the proximal QTL on BTA19 across six cohorts of BBC sires grouped by birth year.

To provide additional evidence that the two genome-wide significant QTL on BTA19 contributed to the increase in muscle mass experienced by BBC since 1985, we examined the evolution of their allelic frequency during the corresponding time lapse. The frequency of the *MRC2 c.2904-2905delAG* variant increased from ~0.02 for bulls born before 1985 to ~0.18 for those born between 2000 and 2004 (implementation of marker assisted selection against the CTS reduced the frequency for bulls born after 2004). For the proximal BTA19 QTL (7.8 Mb), the frequency of the AHAP9 haplotype increased from ~0.17 for bulls born before 1985 to ~0.42 for bulls born during the 2000-2004 interval (Figure 5). To evaluate the significance of these changes, we compared them with the changes undergone during the same period by all variants with a frequency of 0.02 prior to 1985 or haplotypes with a frequency of 0.17 prior to 1985. The increases in frequency of both the *c.2904-2905delAG* variant and AHAP9 haplotype exceeded the corresponding 99th percentiles (the cumulative distribution function equals 0.993 for *c.2904-2905delAG*, and 0.998 for AHAP9*)*, hence suggesting that they were driven by selection.

We next addressed the last question, i.e. ***of the importance of the polygenic or quasi-infinitesimal component of muscularity in BBC***. When adding the MRC2 genotype as fixed effects and the four significant or suggestive QTLs as random effects in a mixed model including a random animal effect, we estimated that the residual polygenic variation accounted for 76% (BM) to 92% (RMR and RMS) of the additive genetic variation. To gain additional insight in the molecular architecture of this polygenic component, we evaluated the performances of “genomic selection” models (including the *MRC2* genotype and the number of AHAP9 copies (at the proximal QTL on BTA19) as fixed effects) differing in their prior assumptions regarding the distribution of polygenic effects. While GBLUP [10] assumes that the effects of all SNPs are drawn from the same normal distribution, BayesB [11] and BayesR [12] assume that the majority of SNPs have no effect, while effects of the remainder are either drawn from student's t-distributions (BayesB) or from a mixture of three normal distribution (BayesR). In a cross-validation design, GBLUP performed as good or better than the Bayesian models (Table 2). This strongly suggests that the residual component of the additive variance is in essence quasi-infinitesimal with a largely uniform distribution of polygenic effect. Indeed, Hayes et al. [13] and Daetwyler et al. [14] showed that for traits with a proportion of moderate to large effects, models such as BayesB tended to perform better than GBLUP.

**Table 2:** **Accuracy of genomic predictions (measured as the squared correlation between predicted breeding values and DTD of 63 bulls born in 2005 or later) obtained with GBLUP, BayesR and BayesB**

Trait	GBLUP	BayesR	BayesB
Back muscling (BM)	0.454	0.447	0.389
Shoulder muscling (SM)	0.490	0.490	0.454
Rump muscling - rear view (RMR)	0.503	0.488	0.418
Rump muscling - side view (RMS)	0.365	0.336	0.287
General muscling (GM)	0.407	0.384	0.226

## Discussion

We herein analyze the genetic underpinnings of the muscular hypertrophy that has continued to augment in BBC after fixation of the *p.D273RfsX13 MSTN* variant around 1985. ***We have not obtained convincing evidence of the fixation of alleles with major effect on muscle mass (other than at the MSTN locus) in modern BBC between 1985 and the present.*** This is unlikely due to a lack of power as three convincing signatures of selection, corresponding to previously described major gene effects, were successfully identified. These involve respectively (i) the fixation of the *MSTN p.D273RfsX13* mutation in (old and new) BBC marked by a ~500 Kb segment of reduced variation and high differentiation, (ii) the fixation of the *MC1R p.L99P* mutation in (old and new) BBC, BBM and HF, underlying the shared, breed-defining dominant black color of the pigmented hairs, and marked by a ~700 Kb segment of reduced variation, (iii) the fixation of a previously characterized haplotype spanning *PLAG1* in (old and new) BBC, BBM and HF, associated with a major positive effect on stature [6], and marked by a ~600 Kb segment of reduced variation. That selection underlies the fixation of the *MSTN p.D273RfsX13* mutation in (old and new) BBC and the *MC1R p.L99P* mutation in (old and new) BBC, BBM and HF is obvious. Selection is also very likely to underlie the segment of reduced genetic variation encompassing *PLAG1*. The present-day standard shoulder height is > 140 cms for several breeds, including HF and BBC, while it was only ~110 cms in the 11^th^ century [15]. Increasing stature has been a declared selection objective in several breeds during the last 50 years as it was a corollary of increased productivity, and this would have expectedly lead to the fixation of the stature-enhancing *PLAG1* allele in these breeds [4, 6]. It is worth noting that the same chromosome region was shown to affect other traits of economic importance, including fertility, food intake or fat depth [16]. These pleiotropic effects might also have contributed to the fixation of the corresponding haplotype. ***We detected two segregating QTL by GWAS, which explain up to 8.5% and 7.2% of genetic variation for muscularity traits, respectively.*** Both QTL are located on BTA19 and appear each to involve one “Q allele” embedded in a unique haplotype (AHAP17 and AHAP9, respectively). The distal BTA19 QTL seems to be largely driven by the *MRC2 c.2904-2905delAG* variant shown previously to cause CTS in homozygotes. Indeed, the *c.2904-2905delAG* variant is in complete LD (D’ ~ 1) with AHAP17, and fitting *c.2904-2905delAG* as a covariate in the model completely annihilates the QTL signal for GM, BM and SM. Independent evidence supporting the causality of the *MRC2* gene are (i) the allelic heterogeneity of CTS in BBC, and (ii) the known role of *MRC2* in bone formation. We detected two independent *MRC2* loss-of-function mutations causing CTS: *c.2904-2905delAG* and *c.1906 T > C* [8, 9]. Such allelic heterogeneity for genetic defects is very unusual in livestock populations that are typically characterized by effective population sizes < 100. We surmise that breeders have unwittingly selected both deleterious *MRC2* mutations because of their visible and desirable effect on muscularity and stature in heterozygotes [8, 9]. *MRC2* is an endocytic collagen receptor that plays an essential role in remodeling the extracellular matrix required during bone growth. Accordingly, the length of long bones, including tibia and femur, is reduced in *MRC2* knock-out mice [17]. That the *MRC2 c.2904-2905delAG* and *c.1906 T > C* directly affect stature is thus very likely. Their apparent effect on muscularity could therefore just reflect a change in the ratio of muscle mass (unchanged) to skeleton size (diminished). Despite the strong evidence supporting the causality of the MRC2 loss-of-function variant, it is worth noting that adding the c.2904-2905delAG in the model did not completely eliminate the QTL effects on RMR and RMS. This suggests that other, as of yet unidentified variants affecting muscularity might exist in this genomic region.

Our molecular understanding of the proximal BTA19 QTL is much more rudimentary. Mining available genome-wide resequencing data for 50 BBC sires (data not shown), did not reveal any striking mutation associated with the AHAP9 haplotype that might underlie the QTL. It is worthwhile noting, however, that the two BTA19 QTL appear to affect different body segments. The distal QTL (47.7 Mb) primarily affects BM and height, while the proximal QTL (7.8 MB) primarily affects RM. This suggests that different molecular pathways are recruited to control the growth of distinct muscle groups.

Assuming that the two BTA19 QTL are genuine, the corresponding “Q” alleles should undergo a selective sweep in the BBC population as a result of the intense selection for increased muscularity. We previously demonstrated that the proportion of present-day offspring of Précieux (the bull that is thought to have introduced the *MRC2 c.2904-2905delAG* variant in the BBC population) is much too large to be explained by Mendelian segregation in the corresponding pedigree alone. Based on these data, we estimated that carriers of the *MRC2 c.2904-2905delAG* had nearly twice as much chance to be selected as elite sires than their non-carrier sibs [8]. In this work, we extended this analysis for the two BTA19 QTL. We compared the magnitude of the increase in frequency between 1985 and 2004 for the *MRC2 c.2904-2905delAG* variant and the AHAP9 haplotype, with that of control variants/haplotypes matched for population frequency in 1985. During this period, the frequency of the *MRC2 c.2904-2905delAG* variant increased by 16% and that of the AHAP9 haplotype by 26%. Both values exceeded the corresponding 99^th^ percentile, hence supporting the selective sweep hypothesis. The 16% increase of the *c.2904-2905delAG* variant is even more remarkable as it is lethal in homozygotes which would have caused counterselection. Further increase of the frequency of the *MRC2 c.2904-2905delAG* variant would probably have stalled, as the proportion of affected homozygotes would have augmented. Such balancing selection now appears to be relatively common in livestock [18]. Paradoxically, knowledge of the underlying causative mutations may lead to the further increase in frequency of the corresponding variants, as it will be possible to avoid at risk matings by marker assisted selection. Unless the AHAP9-associated Q allele of the 7.8 Mb BTA19 QTL also has pleiotropic deleterious effects, and provided that selection for increased muscularity remains the norm, it will likely continue to increase in frequency in BBC until reaching fixation in a few decades. As we have neither observed a depletion in homozygotes for the AHAP9 haplotype, nor detected obvious loss-of-function variants in the immediate vicinity of the 7.8 Mb BTA19 QTL (data not shown), we have no reasons to suspect that such associated deleterious effects might exist.

The fact the “Q” alleles of the two BTA19 QTL were embedded in a single haplotype suggests that the corresponding causative mutations were young and selected for as soon as they appeared in the population. Detectable phenotypic effects of both mutations in heterozygotes would have facilitated the corresponding sweeps. If the corresponding mutations had been old, they would have had time to recombine into multiple haplotype backgrounds. Intense selection for increased muscle mass would then have increased the frequency of the causative mutations, and with these the frequency of not only one (as observed) but of multiple haplotypes. In such case, multiple haplotype states would have been associated with increased muscle mass, rather than one as observed (Figures 3 and 4). An additional argument supporting the young age of the *MRC2 c.2904-2905delAG* variant is its breed specificity: it has only been reported in BBC despite the full sequencing of several hundred individuals representing multiple breeds [19]. We acknowledge that we cannot fully exclude a scenario in which the causative mutations were in fact older, but that as a result of drift (enhanced by artificial insemination) one haplotype only ended up dominating the selective sweep.

One might a priori expect that the suggestive selective sweeps undergone by the AHAP17 and AHAP9 haplotypes would be accompanied by detectable signatures of selection specific for segregating sites (whereas the method implemented in SWEEPY identifies signatures of selection associated with complete sweeps - where the selected variant has reached fixation). To that end, we evaluated the integrated haplotype score (iHS) [20] that searches for haplotypes that are unusually long given their population frequency. The signals obtained in the vicinity of the two BTA19 QTL would not stand above the noise (data not shown). This is likely due to the extensive use of AI, disseminating long-range haplotypes from popular sires in the population even in the absence of direct selection, to strong genetic drift in populations with small effective size or to the selection for other variants across the genome (affecting other selected traits). These findings are in agreement with recent findings from Kemper et al. [21] who reported little evidence for association between strong selective sweeps and regions affecting complex traits under selection. Thus, mapping signatures of selection may not be a very effective alternative to GWAS for the identification of genomic regions influencing economically important traits, as the peculiar structure of livestock populations generates high background.

In addition to these two genome-wide significant BTA19 QTL, we obtained suggestive evidence for three additional QTL for muscularity. Taken together, these five QTL explain between 8.5 and 21.5% of the additive genetic variation. ***We herein provide evidence that the remaining 78.5-91.5% of the additive genetic variation is largely quasi-infinitesimal.*** Indeed, GBLUP (which assumes a uniform distribution of genetic effects across the genome) performed as well as Bayesian models (which allow for an exponential distribution of gene effects) in predicting muscularity in cross-validation experiments. We attempted to characterize the polygenic architecture of muscularity from the direct output of the Bayesian models (f.i. proportion π of SNPs with an effect in BayesCπ [22], or number of SNPS in the four sub-populations in BayesR [12]). As the corresponding numbers did not differ significantly from those obtained with permuted phenotypes, they were considered unreliable with our limited data set (data not shown), and cross-validation used instead.

Thus, the genetic architecture of muscularity in modern BBC – a typical production trait subject to intense directional selection – involves (i) a handful of detectable QTL that individually explain of the order of 5%, and jointly of the order of 8.5 to 21.5% of the additive genetic variance (the *p.D273RfsX13 MSTN* variant has an even larger effect; it has been fixed in a few generations and does not contribute any longer to the genetic variation in modern BBC), and (ii) a quasi-infinitesimal polygenic tail of what are likely to be a very large number of variants with very small effects that would require immense sample size to be individualized. Moreover, the data suggests that the detectable QTL might correspond to novel variants that appeared only recently in the population. Evidence from QTL analyses of other production traits in livestock, including milk production and composition in cattle [23], body size in cattle [6], muscularity in pigs [24] and sheep [25, 26], fertility in sheep [27, 28], etc., suggests that the same may apply to all these phenotypes. This “bivariate” composition, also pointed out by Kemper and Goddard [29], seems to be in sharp contrast with findings from GWAS conducted in humans. Indeed, nearly all studied complex traits in humans appear to only comprise the quasi-infinitesimal/polygenic component (e.g. [30, 31]). QTL accounting for 5% of the additive genetic variance are essentially unheard off in human genetics. On the contrary the adaptation of threespine sticklebacks to marine and freshwater environments [32] appears also to have involved a small number of genes with large effects, a situation that appears reminiscent of the highly selected livestock populations. The biological reasons underlying this dichotomy remain largely unexplained, but it is tempting to speculate that it is related to the strong directional selection that applies to livestock populations and – in specific circumstances - to natural populations. Our results support a model in which response to directional selection in domesticated species, involves (i) the rapid fixation (“hard sweep”) of a small number of variants with moderate to large phenotypic effects that sequentially arise in the population by mutation and may jointly account for > 10% of the genetic variance, and (ii) slow and gradual changes in frequencies (“polygenic adaptation”) of a large pool of variants with very small phenotypic effects, of which most have been segregating in the population for a long time (“standing variation”) and jointly account for the bulk of the genetic variance.

## Conclusions

Contrary to complex traits in humans which have a near-exclusively polygenic architecture, muscle mass in beef cattle (as other production traits under directional selection), appears to be controlled by (i) a handful of recent mutations with large effect that rapidly sweep through the population, and (ii) a large number of presumably older variants with very small effects that rise slowly in the population (polygenic adaptation).

## Methods

### Data

A set of 593 Belgian-Blue beef (BBC), 52 Belgian-Blue dual-purpose (BBM) and 191 Dutch Holstein (HF) bulls were genotyped with the BovineHD genotyping array (Illumina, San Diego, CA) containing 735,293 SNPs mapping to autosomal chromosomes (on the UMD3.1 built). Individuals were required to have a call rate above 0.90.

For identification of selective sweeps, a subset of 301 BBC sires born in 2000 or later (after 30-40 years of intense selection for muscular development) were representing ‘modern’ BBC whereas 28 BBC sires born in 1985 or earlier represented ‘old’ BBC. Markers with call rates below 95% in any of the three breeds or significantly deviating from HWE (p < 0.001) in at least one breed were excluded from the study. As a result, 672,754 SNPs were conserved.

### Phenotypes

For association studies and genomic prediction, we used the genetic evaluations of Belgian-Blue sires for five muscularity traits (back muscularity (BM), shoulder muscularity (SM), rump muscularity - rear view (RMR), rump muscularity - side view (RMS) and a synthetic note of muscularity (GM) obtained by a linear combination of the previous traits (GM = 1 × SM + 1 × BM + 2 × RMR + 2 × RMS)). For individual traits, the animals were scored from 1 to 50 by a technician. These conformation traits were measured on their daughters between 15 and 56 months of age. The phenotypic values were equivalent to daughter-trait deviations (DTD) as proposed by VanRaden and Wiggans [33]. For each sire, the DTD is a weighted mean of the phenotypic records of their daughters corrected for fixed effects and half of the genetic value of the mate. As described in VanRaden and Wiggans [33], each DTD is weighted by a number of effective daughters or daughter equivalents (DE). DTD scale like estimated transmitting abilities (ETA) and we used twice the DTD (scaling as estimated breeding values – EBV).

### Identification of regions potentially associated with complete sweeps

We used SWEEPY [4] to identify regions of reduced heterozygosity for a long stretch of markers. The method relies on a HMM with three hidden states corresponding to 1) regions of reduced heterozygosity (called “sweep” regions), 2) background or neutral regions and 3) intermediate regions (transitions from “sweep” to “background” states or vice versa require going through this intermediate state). Each hidden state is defined with its emission probabilities (the probability to observe a marker with heterozygosity *h* within a given hidden state). For “sweep” regions, the distribution of heterozygosity maximizes probability of low heterozygosity and penalizes markers with moderate or high levels of heterozygosity. For the intermediate and the neutral state, the emission probabilities were estimated from the data (see [4] for more details).

This HMM was applied to the ‘modern’ BBC. Regions for which the probability to be in the “sweep” state were higher than 0.5 were then identified. Regions where none of the markers reached a ‘sweep probability’ above 0.9999 were discarded. In addition, regions with average marker density below 100 SNPs/Mb were excluded from further investigation.

Only regions presenting differentiation with HF, BBM or ‘old’ BBC were conserved since we focus on recent selective sweeps associated with muscular development, which should be specific to the modern BBC breed. To that end we computed F_ST_ measures [34] between “modern” BBC and HF, BBM or “old” BBC. Potential sweep regions were considered differentiated with one of the three control populations if the average F_ST_ value (for the SNPs in the segment) with that population was higher than the 95^th^ percentile of the average F_ST_ values (with the same population) for a set of 10,000 randomly sampled regions containing the same number of SNPs.

### GWAS

Association analyses were conducted using a previously described haplotype-based method that corrects for stratification by means of a random polygenic effect [7]. Haplotypes (obtained with Beagle [35]) are assigned to K = 20 ancestral haplotypes (cluster of similar haplotypes) with PHASEBOOK [7] and fitted in the following mixed model:


where **y** is a vector of DTD, **h** is the vector of random QTL effects corresponding to the K defined ancestral haplotypes, **Z**_**h**_ is an incidence matrix relating ancestral haplotype effect to records of sires, **u** is the vector of random individual polygenic effects (e.g., [36]), **Z**_**u**_ is an incidence matrix relating polygenic effects to records of sire and **e** is the vector of individual error terms. Ancestral haplotypes effects with corresponding variance , polygenic effect with corresponding covariance structure  (with **A** the additive relationship matrix estimated from available genealogical information) and individual error terms with corresponding variance  (w_i_ is the weight corresponding to DTD of individual i and corresponds to the number of records equivalents as estimated in VanRaden and Wiggans [33] or Garrick et al. [37] were estimated by Average Information Restricted Maximum Likelihood (AI-REML) analysis [38]. Evidence for the presence of a QTL was measured by a likelihood ratio test (LRT) comparing the likelihood of the data assuming a model with versus without a haplotype effect at the interrogated map position. The LRT statistic was assumed to be distributed with a chi-square distribution with one df.

Markers with call rates below 95% in the 593 BBC bulls or significantly deviating from HWE (p < 0.001) were not used for association studies or genomic prediction. As a result, a subset of 708,579 SNPs was used.

### Correction for multiple-testing

Genome-wide significance thresholds at p < 0.05 were defined based on Bonferroni corrections for multiple-testing with N independent tests. Suggestive QTL correspond to level of significance which are expected to be reached once per genome scan and correspond to a threshold of p <0.37 (e.g. [39]). The number of independent tests is a function of the number of tested SNPs and their correlation (due to the linkage disequilibrium). We estimated the number of independent tests using the following approach. First, we estimated - for each SNP - the p-value for association with one of the studied traits (for instance GM) based on a simple ANOVA test. These correspond to uncorrected p-values (α). Next, we performed 10,000 random permutations of the phenotypes and – each time - scanned the entire genome (i.e. tested all SNP) for association with the permuted phenotype. For each permutated data set, we kept the “best p-value” obtained across all the tested SNP (i.e. across the entire genome). This provided a distribution of “best p-values” across the genome for 10,000 permuted data sets, hence under the null hypothesis that no QTL exist. We used this set of “best p-values” (obtained with the permuted phenotypes) to determine – for each SNP – a p-value of association (with the real phenotypes), corrected for the analysis of the whole genome (or α*). This corrected p-value α* corresponds to its rank with respect to the sorted list of “best p-values” obtained with the permuted data. This yielded two p-values of association (α and α*) for each SNP. From these lists we estimated the number of independent tests, *N,* by simple linear regression, realizing that:


We only used corrected p-values ranging between the 1^th^ and 99^th^ percentiles for this analysis.

Using this approach, we estimated that the number of independent tests was equal to 20,130 (rounded to 20,000) and the resulting genome-wide significance threshold was fixed at 5.60 (4.73 for suggestive QTLs).

### Genomic predictions

We applied three models to perform genomic predictions that fit all the SNP simultaneously. The first model is the so-called GBLUP [10]:


where **y** is a vector of records (DTD), **X** is an incidence matrix relating fixed effects to records (it includes a variable indicating whether the individual is carrier of the Crooked-Tail Syndrome (CTS) mutation and another variable equal to the number of copies of haplotype AHAP9 (see Results) carried by the individual), **b** is a vector of fixed effects including the mean, the effect of the CTS mutation and of one copy of haplotype AHAP9, **g** is a vector of individual genomic (or polygenic) effects with variance  (**G** being the genomic relationship matrix), **Z**_**g**_ is an incidence matrix relating individuals to respective records and **e** is the vector of individual error terms with variance . The genomic relationship matrix was obtained as (e.g., [10, 12]):


With *f*_*k*_ being the frequency of allele 1 for marker k and **W** is an N_IND_ x N_SNP_ matrix where element w_ik_ is the number of alleles 1 for SNP k for individual i minus twice *f*_*k*_ .

Individual genomic effects (**g**) for animals with and without phenotypes and variance parameters  and  were estimated with AI-REML analysis [38].

The second model is a Bayesian model called BayesB [11]:


Where **z**_k_ is a N_IND_ x 1 vector of allelic counts (number of alleles 1) for SNP k, v_k_ is the allelic substitution effect for SNP k, **Z**_**v**_ is a N_IND_ × N_SNP_ matrix with column k equal to **z**_k_ and **v** is the N_IND_ × 1 vector of allelic substitution effects. Individual genomic effects can be obtained as **Z**_**v**_**v**.

The prior for the allelic substitution effects are (e.g., [22]):


Where π is the probability of SNP having a non-zero effect and is a parameter related to the number of QTL (or the proportion of fitted QTLs relative to the total number of SNP). As suggested by Garrick and Fernando [40], the parameter π was estimated with the model BayesCπ implemented in GenSelR [40]. The variance of the allelic substitution effect is modeled as an inverse chi-square distribution:


The BayesB model was solved by MCMC techniques using GenselR.

The third model is BayesR and includes fixed effects, polygenic effects (with the relationship matrix obtained from genealogical information) and SNP effects [12, 41]:


SNP effects are normally distributed  and the variance of *i*th SNP effect has four possible values . All parameters were estimated (including proportion of SNPs in each variance class) as described in Erbe et al. [12] and Kemper et al. [41] with BayesR. As in the GBLUP model, the residual variance was weighted in both Bayesian models.

SNPs used in the association study were further filtered. First, SNPs with a MAF below 0.01 were discarded. Then, SNPs were clustered based on linkage disequilibrium: SNP were added to a cluster if they presented a r^2^ value above 0.95 with at least one SNP from the cluster. Only one SNP per cluster was conserved to reduce the number of highly correlated variables and reduce confounding effects. As a result, only 289,707 SNPs were selected.

The efficiency of genomic prediction was assessed by splitting the data in two groups: a set of 492 animals born prior to 2005 and 63 bulls born after January 1^st^ 2005. The phenotypes from these 63 more recent bulls were erased and not used in the genomic prediction model. Then, the accuracy of genomic prediction was estimated as the squared weighted correlation between predicted genomic values (including fixed effects of the MRC2 genotype and the AHAP9) and DTD (using the number of effective records as weights).

### Ethics statement

No animal experiments were performed specifically for this manuscript. Where data were obtained from existing sources, references for these experiments are provided.

## Electronic supplementary material

Additional file 1: Figure S1: Comparison of carcass scores in function of the *mh* mutation. Carcass scores of BBC (which are all homozygous for the *p.D273RfsX13 MSTN* mutation or *mh/mh* (red)), and dual-purpose BBM cows (which can be either *mh/mh* (orange), *mh/+* (yellow), or *+/+* (white). The effect of the partial recessive *mh* allele can be seen from the superiority of *mh/mh* over *mh/+* and +/+ BBM animals. The additional superiority of *mh/mh* BBC over *mh/mh* BBM animals highlights the effects of other muscle-enhancing genetic variants in BBC animals. (TIFF 950 KB)

Additional file 2: Figure S2: Measures of genetic distances between different bovine breed samples genotyped with a Bovine 50 K chip. Each breed is represented with a different color (orange for Belgian Blue beef cattle, blue for Belgian Blue dual-purpose cattle and magenta for Holstein). BBB = Belgian-Blue beef, BBM = Belgian -Blue dual-purpose, HOL = Holstein, ANG = Angus, CHL = Charolais, SIM = Simmental, JER = Jersey, HRF = Hereford, WAG = Wagyu, SEP = Senepol, SGT = Santa-Gertrudis, BRM = Brahman, NEL = Nelore. A. Coordinates (first two coordinates) obtained from a mutli-dimensional scaling analysis. B. Neighbourgh joining tree. Distance was measured based on number of identical-by-state allele at each marker allele. (ZIP 10 KB)

Additional file 3: Table S1: Table reporting the 91 candidate regions exhibiting reduced variability in modern BBC and identified with SWEEPY. (XLSX 15 KB)

Additional file 4: Figure S3: Description of the sweep encompassing *MSTN*. The top panel represents the sweep probability estimated by Sweepy (red curve), the SNP heterozygosity in BBC (grey curve), the differentiation (measured as F_ST_) with BBM (orange points) and HF (blue points). The lower panel represents the local Ensembl annotation. (TIFF 3 MB)

Additional file 5: Figure S4: Description of the sweep encompassing *PLAG1*. The top panel represents the sweep probability estimated by Sweepy (red curve), the SNP heterozygosity in BBC (grey curve), the differentiation (measured as F_ST_) with BBM (orange points) and HF (blue points). The lower panel represents the local Ensembl annotation. (TIFF 2 MB)

Additional file 6: Figure S5: Description of the sweep encompassing *MC1R*. The top panel represents the sweep probability estimated by Sweepy (red curve), the SNP heterozygosity in BBC (grey curve), the differentiation (measured as F_ST_) with BBM (orange points) and HF (blue points). The lower panel represents the local Ensembl annotation. (TIFF 2 MB)

Additional file 7: Figure S6: Manhattan plots for general muscularity (GM). Alternating gray and black symbols mark the limits between successive chromosomes. The two red horizontal lines correspond to the thresholds for genome-wide significant and suggestive association, respectively. A. Manhattan plot without the *MRC2* genotype in the model. B. Manhattan plot with the *MRC2* genotype in the model. (TIFF 193 KB)

Additional file 8: Figure S7: Manhattan plots for muscularity of the back (BM). Alternating gray and black symbols mark the limits between successive chromosomes. The two red horizontal lines correspond to the thresholds for genome-wide significant and suggestive association, respectively. A. Manhattan plot without the *MRC2* genotype in the model. B. Manhattan plot with the *MRC2* genotype in the model. (TIFF 192 KB)

Additional file 9: Figure S8: Manhattan plots for muscularity of the shoulder (SM). Alternating gray and black symbols mark the limits between successive chromosomes. The two red horizontal lines correspond to the thresholds for genome-wide significant and suggestive association, respectively. A. Manhattan plot without the *MRC2* genotype in the model. B. Manhattan plot with the *MRC2* genotype in the model. (TIFF 190 KB)

Additional file 10: Figure S9: Manhattan plots for muscularity of the rump - rear view (RMR). Alternating gray and black symbols mark the limits between successive chromosomes. The two red horizontal lines correspond to the thresholds for genome-wide significant and suggestive association, respectively. A. Manhattan plot without the *MRC2* genotype in the model. B. Manhattan plot with the *MRC2* genotype in the model. (TIFF 194 KB)

Additional file 11: Figure S10: Manhattan plots for muscularity of the rump - side view (RMS). Alternating gray and black symbols mark the limits between successive chromosomes. The two red horizontal lines correspond to the thresholds for genome-wide significant and suggestive association, respectively. A. Manhattan plot without the *MRC2* genotype in the model. B. Manhattan plot with the *MRC2* genotype in the model. (TIFF 195 KB)

Additional file 12: Table S2: Estimated effect (and associated variance) of the Crooked-Tail Syndrome variant for the five muscularity traits. (DOCX 14 KB)

Additional file 13: Figure S11: Association on BTA19 for general muscularity (GM). The association was performed with a mixed model including a polygenic effect (accounting for stratification or familial relationship), haplotype effects and without (blue) or with (green) correction for the *MRC2* genotypes. The two red horizontal lines correspond to the thresholds for genome-wide significant and suggestive association, respectively. (ZIP 38 KB)

Additional file 14: Figure S12: Association on BTA19 for muscularity of the back (BM). The association was performed with a mixed model including a polygenic effect (accounting for stratification or familial relationship), haplotype effects and without (blue) or with (green) correction for the *MRC2* genotypes. The two red horizontal lines correspond to the thresholds for genome-wide significant and suggestive association, respectively. (ZIP 38 KB)

Additional file 15: Figure S13: Association on BTA19 for muscularity of the shoulder (SM). The association was performed with a mixed model including a polygenic effect (accounting for stratification or familial relationship), haplotype effects and without (blue) or with (green) correction for the *MRC2* genotypes. The two red horizontal lines correspond to the thresholds for genome-wide significant and suggestive association, respectively. (ZIP 38 KB)

Additional file 16: Figure S14: Association on BTA19 for muscularity of the rump - rear view (RMR). The association was performed with a mixed model including a polygenic effect (accounting for stratification or familial relationship), haplotype effects and without (blue) or with (green) correction for the *MRC2* genotypes. The two red horizontal lines correspond to the thresholds for genome-wide significant and suggestive association, respectively. (ZIP 38 KB)

Additional file 17: Figure S15: Association on BTA19 for muscularity of the rump - side view (RMS). The association was performed with a mixed model including a polygenic effect (accounting for stratification or familial relationship), haplotype effects and without (blue) or with (green) correction for the *MRC2* genotypes. The two red horizontal lines correspond to the thresholds for genome-wide significant and suggestive association, respectively. (ZIP 37 KB)

## Abbreviations

AI: Artificial insemination
AI-REML: Average information restricted maximum likelihood
ANOVA: Analysis of variance
BBC: Belgian blue cattle
BBM: Blancs bleus mixtes
BM: Muscularity of the back
CTS: Crooked-tail syndrome
DE: Daughter equivalents
DTD: Daughter trait deviations
EBV: Estimated breeding values
ETA: Estimated transmitting abilities
GBLUP: Genomic best linear unbiased predictor
GM: General muscularity
GWAS: Genome-wide association study
HF: Holstein-Friesian
HMM: Hidden markov model
HWE: Hardy-Weinberg equilibrium
iHS: Integrated haplotype score
LRT: Likelihood ratio test
MAF: Minor allele frequency
MCMC: Markov chain Monte Carlo
MSTN: Myostatin
QTL: Quantitative trait locus
RMR: Muscularity of the rump (rear view)
RMS: Muscularity of the rump (side view)
SM: Muscularity of the shoulder
SNP: Single nucleotide polymorphism.
