# Supplementary figures and images for: Selection in action: dissecting the molecular underpinnings of the increasing muscle mass of Belgian Blue Cattle
Source: BMC Genomics. 2014 Sep 17;15(1):796. doi: 10.1186/1471-2164-15-796 (PMC4190573; doi:10.1186/1471-2164-15-796)

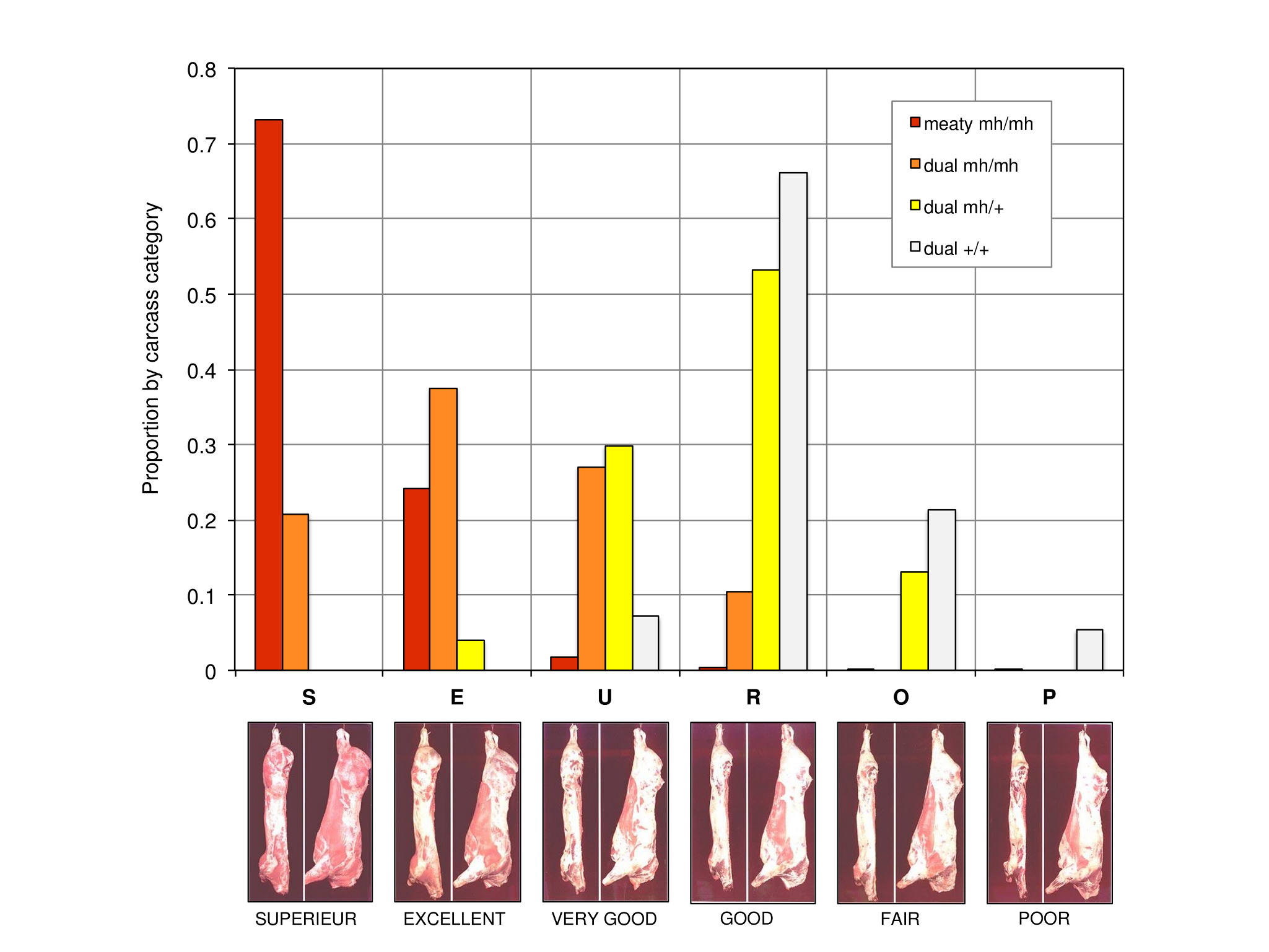

Supplement: Supplementary file 1 — Additional file 1: Figure S1: Comparison of carcass scores in function of the mh mutation. Carcass scores of BBC (which are all homozygous for the p.D273RfsX13 MSTN mutation or mh/mh (red)), and dual-purpose BBM cows (which can be either mh/mh (orange), mh/+ (yellow), or +/+ (white). The effect of the partial recessive mh allele can be seen from the superiority of mh/mh over mh/+ and +/+ BBM animals. The additional superiority of mh/mh BBC over mh/mh BBM animals highlights the effects of other muscle-enhancing genetic variants in BBC animals. (TIFF 950 KB) [file 12864_2014_6507_MOESM1_ESM.tiff]

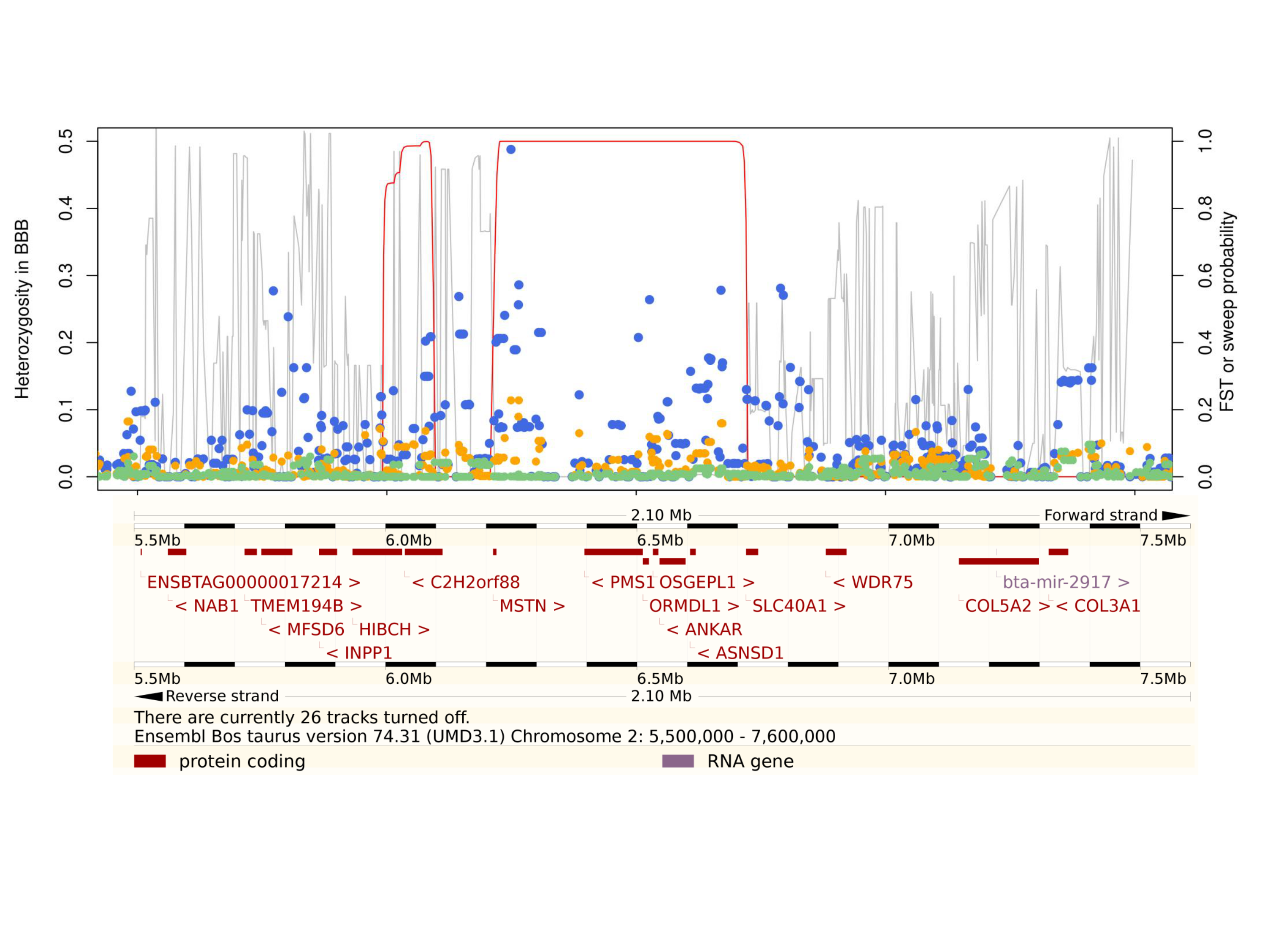

Supplement: Supplementary file 4 — Additional file 4: Figure S3: Description of the sweep encompassing MSTN. The top panel represents the sweep probability estimated by Sweepy (red curve), the SNP heterozygosity in BBC (grey curve), the differentiation (measured as FST) with BBM (orange points) and HF (blue points). The lower panel represents the local Ensembl annotation. (TIFF 3 MB) [file 12864_2014_6507_MOESM4_ESM.tiff]

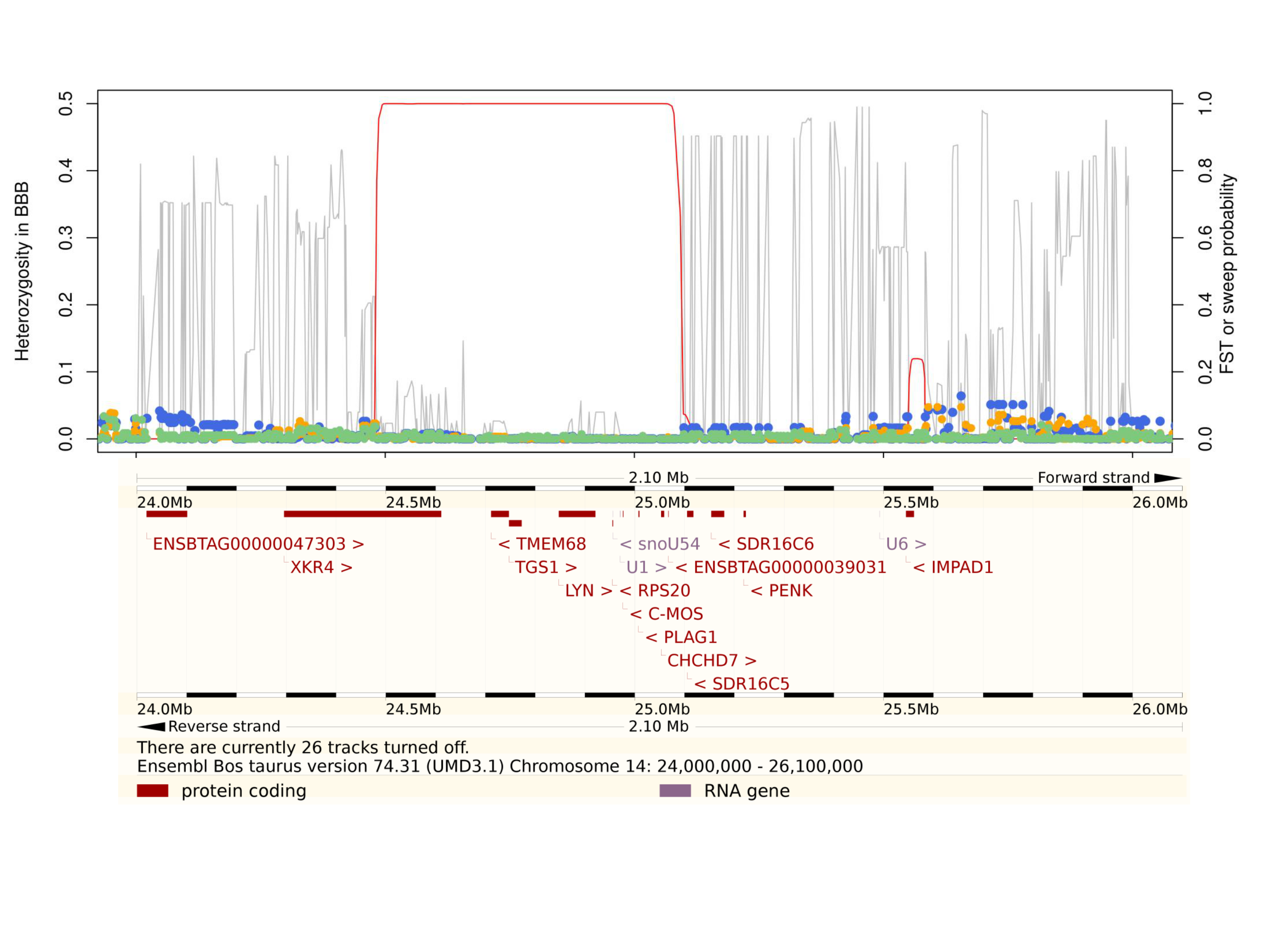

Supplement: Supplementary file 5 — Additional file 5: Figure S4: Description of the sweep encompassing PLAG1. The top panel represents the sweep probability estimated by Sweepy (red curve), the SNP heterozygosity in BBC (grey curve), the differentiation (measured as FST) with BBM (orange points) and HF (blue points). The lower panel represents the local Ensembl annotation. (TIFF 2 MB) [file 12864_2014_6507_MOESM5_ESM.tiff]

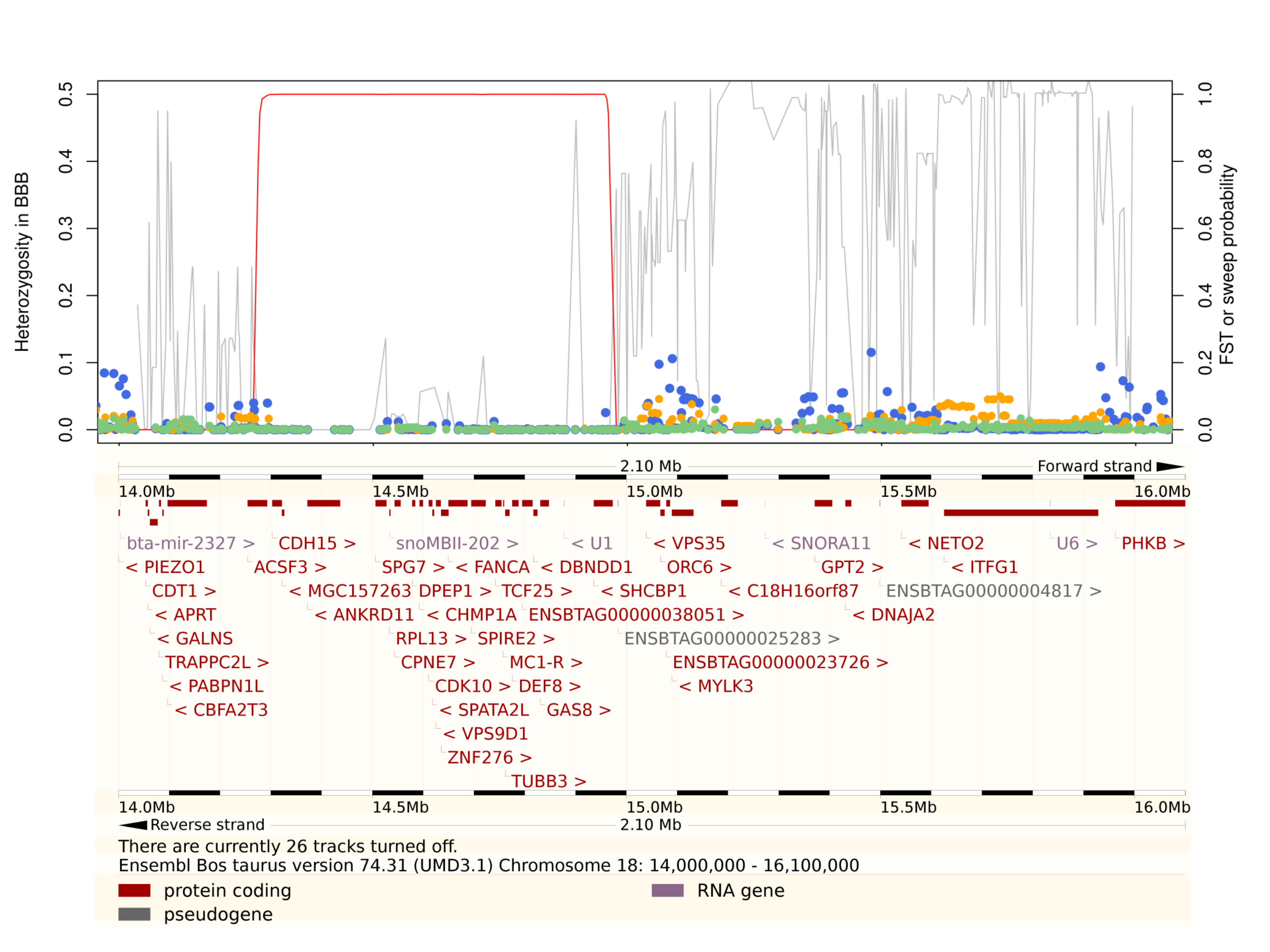

Supplement: Supplementary file 6 — Additional file 6: Figure S5: Description of the sweep encompassing MC1R. The top panel represents the sweep probability estimated by Sweepy (red curve), the SNP heterozygosity in BBC (grey curve), the differentiation (measured as FST) with BBM (orange points) and HF (blue points). The lower panel represents the local Ensembl annotation. (TIFF 2 MB) [file 12864_2014_6507_MOESM6_ESM.tiff]

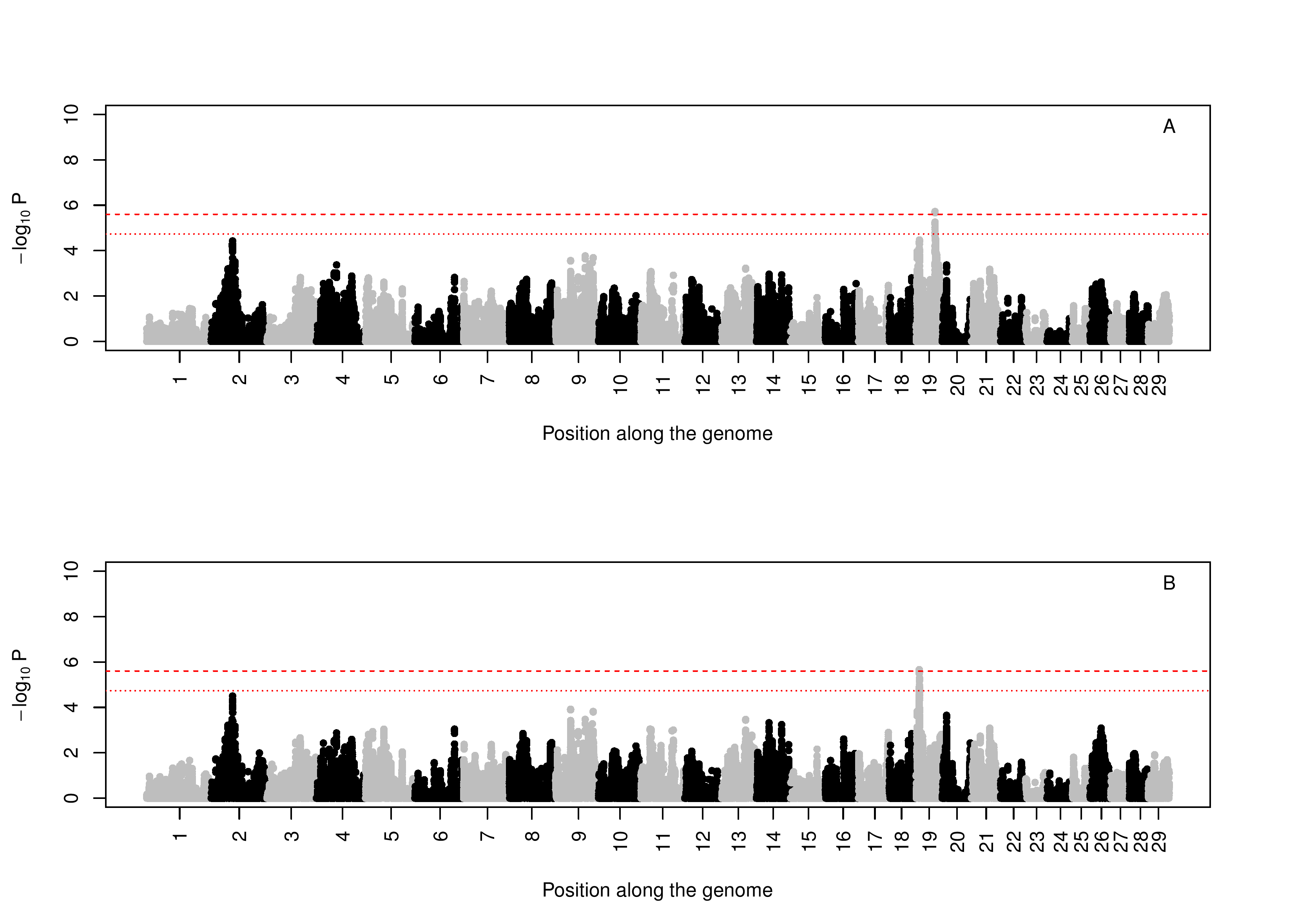

Supplement: Supplementary file 7 — Additional file 7: Figure S6: Manhattan plots for general muscularity (GM). Alternating gray and black symbols mark the limits between successive chromosomes. The two red horizontal lines correspond to the thresholds for genome-wide significant and suggestive association, respectively. A. Manhattan plot without the MRC2 genotype in the model. B. Manhattan plot with the MRC2 genotype in the model. (TIFF 193 KB) [file 12864_2014_6507_MOESM7_ESM.tiff]

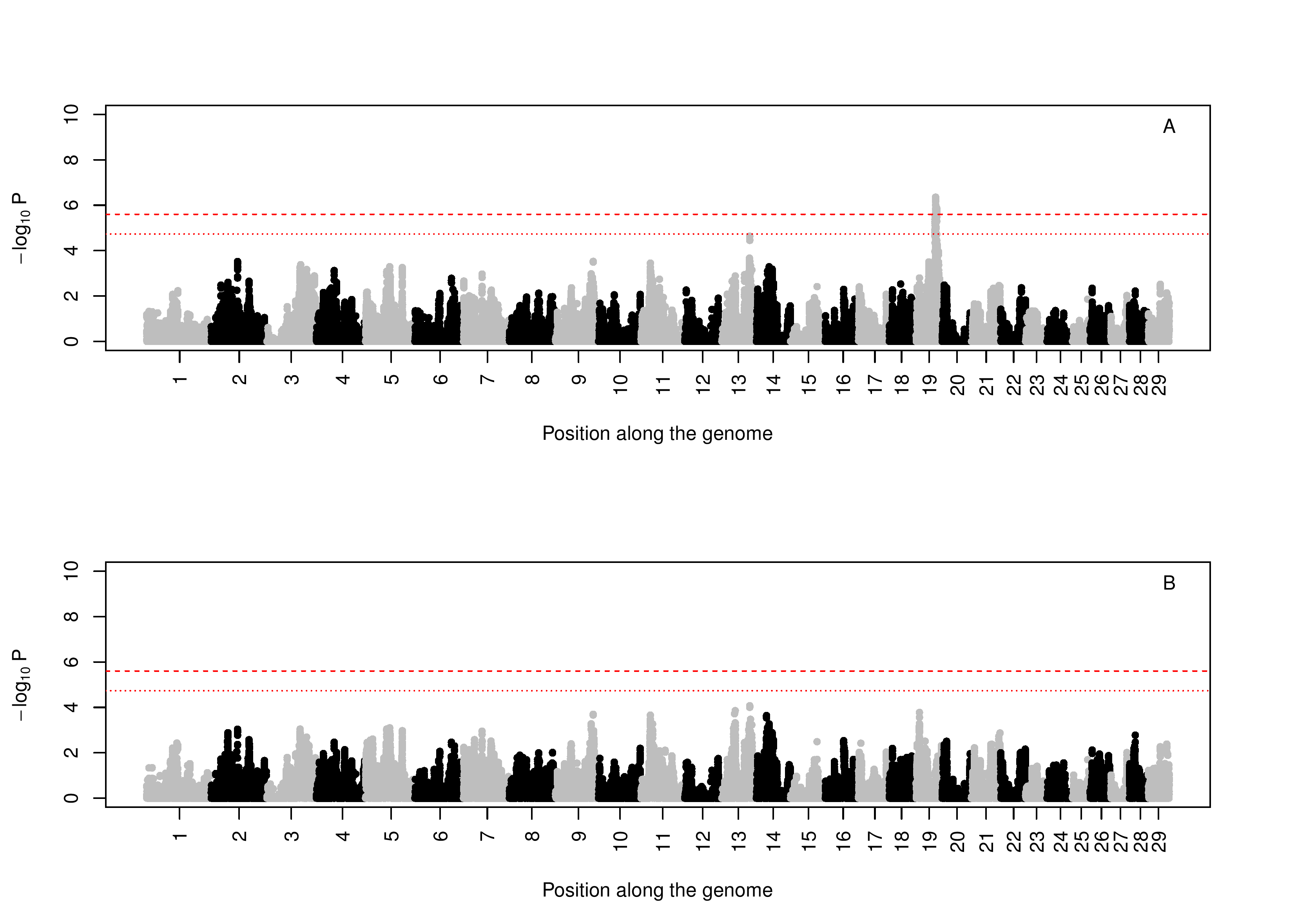

Supplement: Supplementary file 8 — Additional file 8: Figure S7: Manhattan plots for muscularity of the back (BM). Alternating gray and black symbols mark the limits between successive chromosomes. The two red horizontal lines correspond to the thresholds for genome-wide significant and suggestive association, respectively. A. Manhattan plot without the MRC2 genotype in the model. B. Manhattan plot with the MRC2 genotype in the model. (TIFF 192 KB) [file 12864_2014_6507_MOESM8_ESM.tiff]

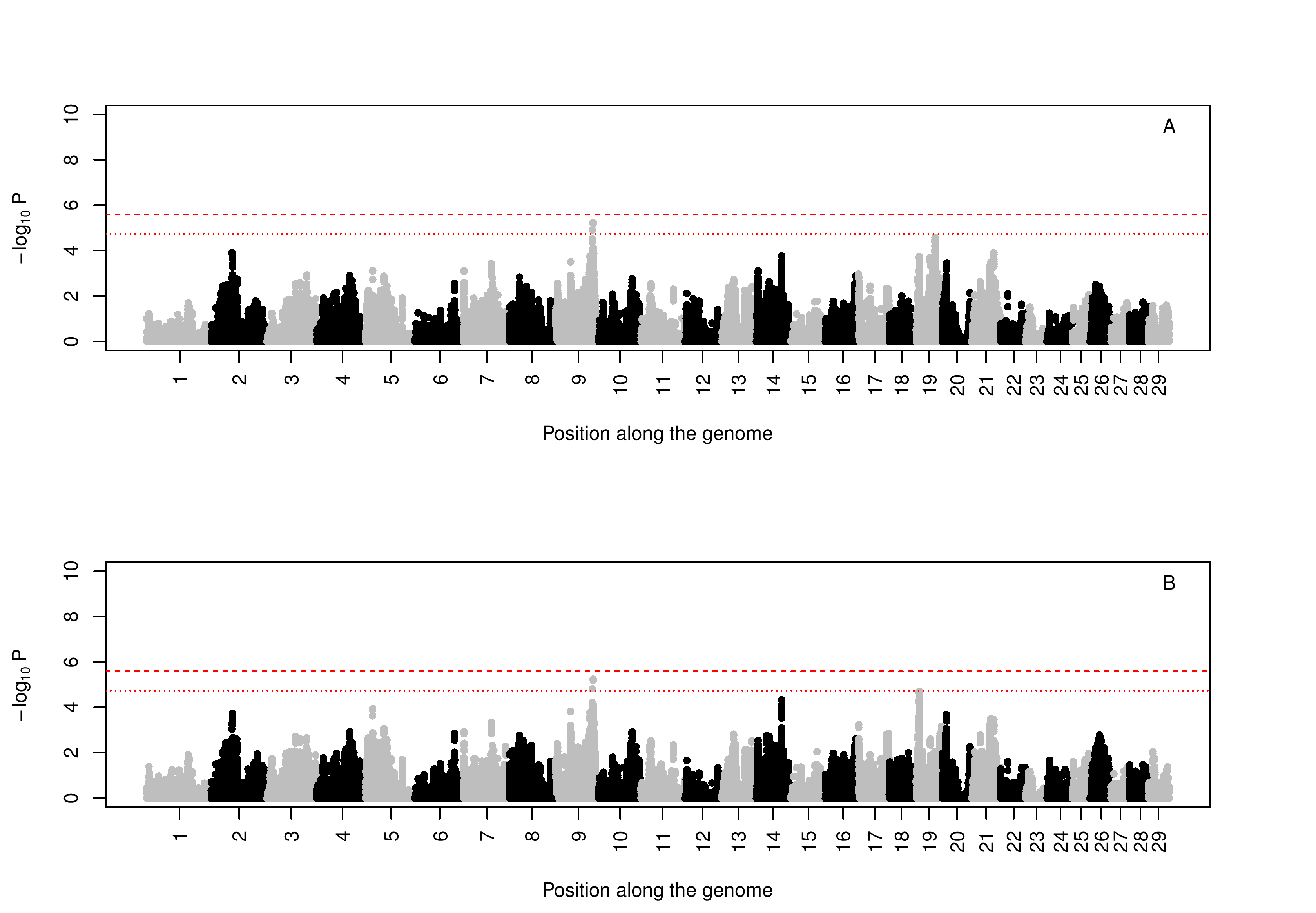

Supplement: Supplementary file 9 — Additional file 9: Figure S8: Manhattan plots for muscularity of the shoulder (SM). Alternating gray and black symbols mark the limits between successive chromosomes. The two red horizontal lines correspond to the thresholds for genome-wide significant and suggestive association, respectively. A. Manhattan plot without the MRC2 genotype in the model. B. Manhattan plot with the MRC2 genotype in the model. (TIFF 190 KB) [file 12864_2014_6507_MOESM9_ESM.tiff]

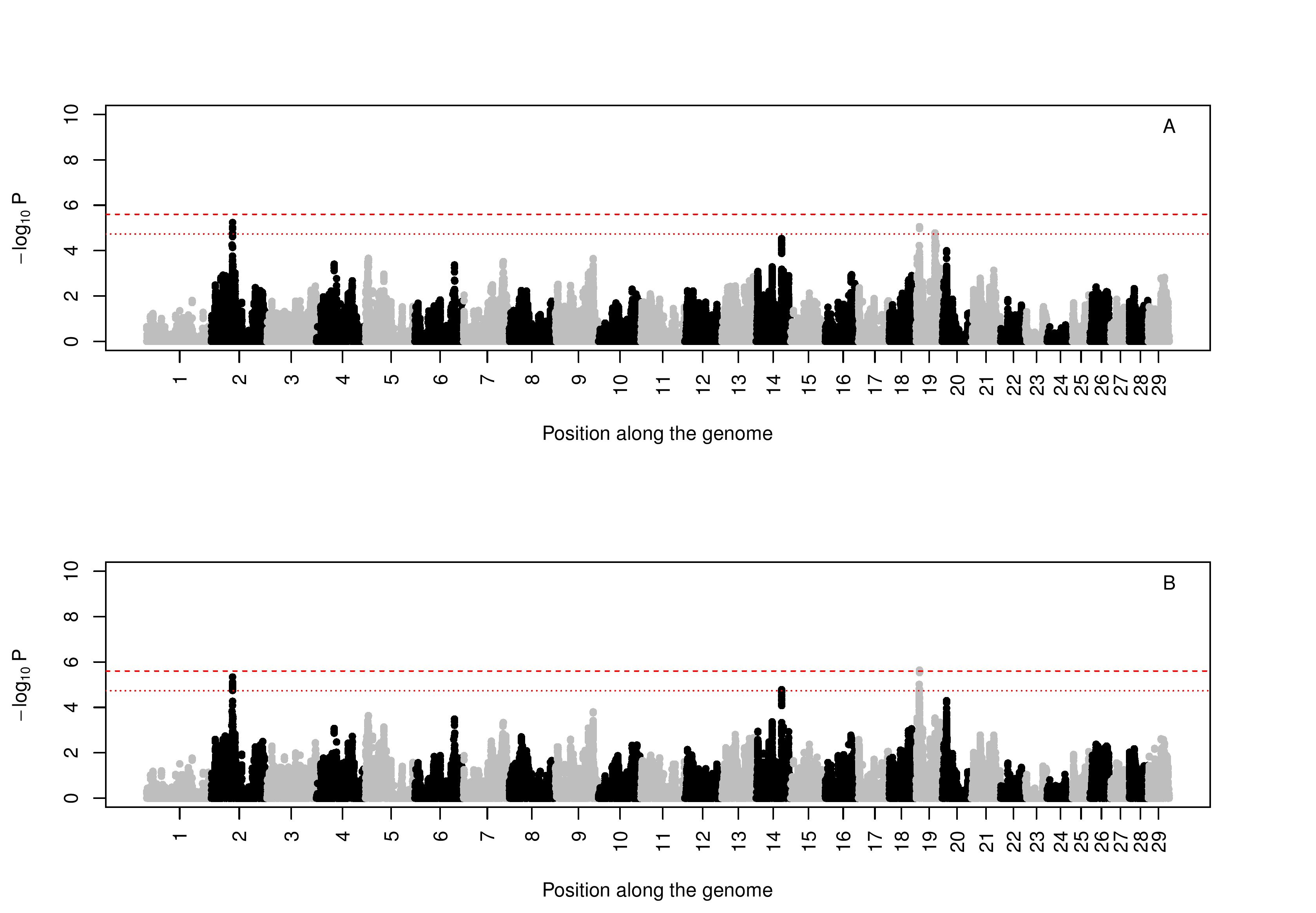

Supplement: Supplementary file 10 — Additional file 10: Figure S9: Manhattan plots for muscularity of the rump - rear view (RMR). Alternating gray and black symbols mark the limits between successive chromosomes. The two red horizontal lines correspond to the thresholds for genome-wide significant and suggestive association, respectively. A. Manhattan plot without the MRC2 genotype in the model. B. Manhattan plot with the MRC2 genotype in the model. (TIFF 194 KB) [file 12864_2014_6507_MOESM10_ESM.tiff]

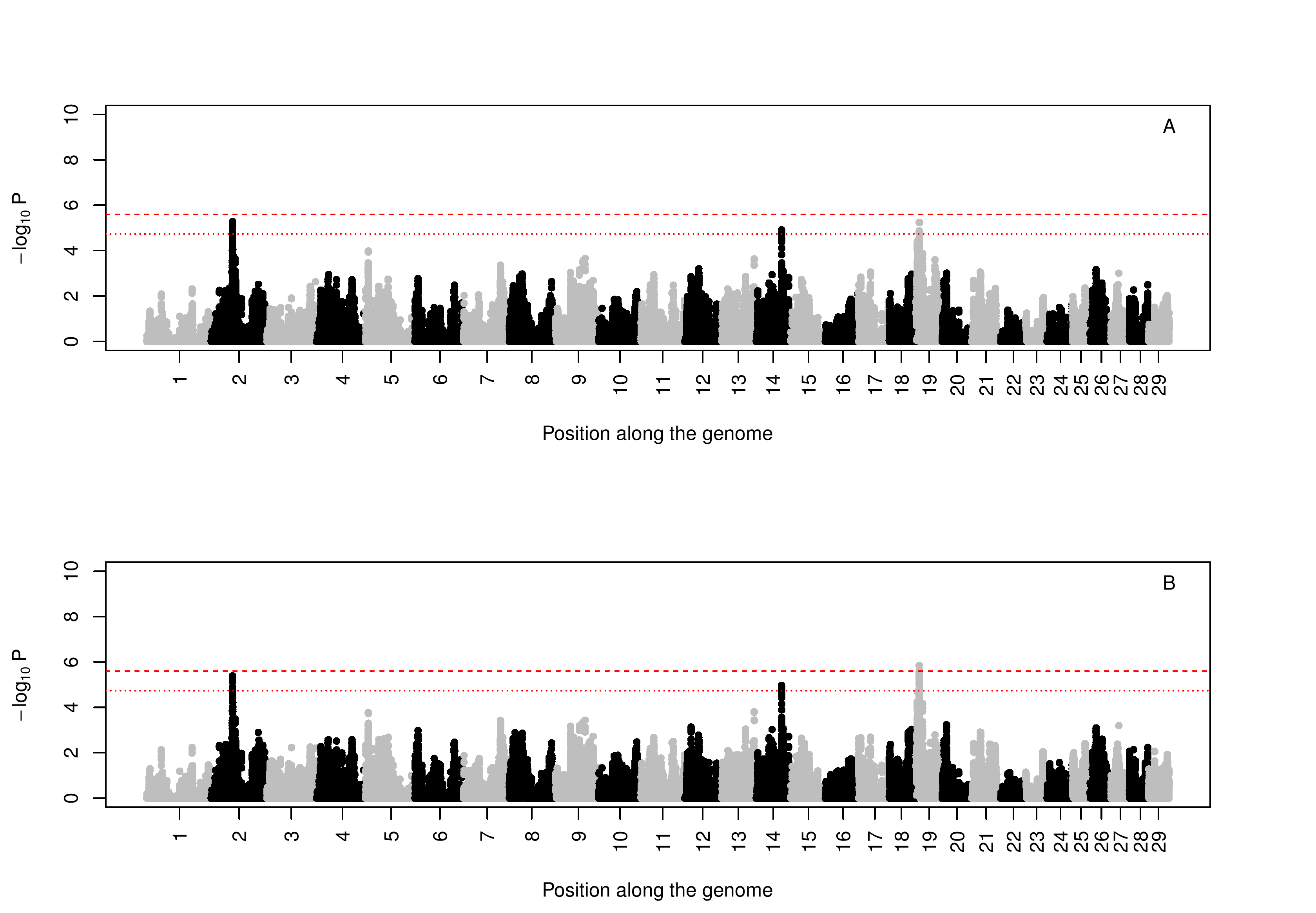

Supplement: Supplementary file 11 — Additional file 11: Figure S10: Manhattan plots for muscularity of the rump - side view (RMS). Alternating gray and black symbols mark the limits between successive chromosomes. The two red horizontal lines correspond to the thresholds for genome-wide significant and suggestive association, respectively. A. Manhattan plot without the MRC2 genotype in the model. B. Manhattan plot with the MRC2 genotype in the model. (TIFF 195 KB) [file 12864_2014_6507_MOESM11_ESM.tiff]
