# Supplementary material for: Selection in action: dissecting the molecular underpinnings of the increasing muscle mass of Belgian Blue Cattle
Source: BMC Genomics. 2014 Sep 17;15(1):796. doi: 10.1186/1471-2164-15-796 (PMC4190573; doi:10.1186/1471-2164-15-796)
Supplement: Supplementary file 12 — Additional file 12: Table S2: Estimated effect (and associated variance) of the Crooked-Tail Syndrome variant for the five muscularity traits. (DOCX 14 KB) [file 12864_2014_6507_MOESM12_ESM.docx]

Additional file 2: Table S2. Effect of Crooked-Tail Syndrome mutations on muscularity traits.

| Trait | Effect^1^ | %Var^2^ | p-value | -Log10(p) |
| --- | --- | --- | --- | --- |
| Back muscularity (BM) | 0.68 | 8.55 | 1.13e-9 | 8.95 |
| Shoulder muscularity (SM) | 0.52 | 4.94 | 7.59e-6 | 5.12 |
| Rump muscularity -rear view (RMR) | 0.39 | 2.83 | 3.34e-4 | 3.48 |
| Rump muscularity - side view (RMR) | 0.32 | 1.95 | 3.94e-3 | 2.40 |
| General muscularity (GM) | 0.56 | 5.92 | 1.86e-6 | 5.73 |

^1^ Divided by genetic standard deviation

^2^ % of genetic variance due to the variant (frequency of 0.24)
